# BET inhibitor JQ1 suppresses cell proliferation via inducing autophagy and activating LKB1/AMPK in bladder cancer cells

**DOI:** 10.1002/cam4.2385

**Published:** 2019-06-28

**Authors:** Feng Li, Chao Yang, Hai‐Bao Zhang, Jianbin Ma, Jing Jia, Xiaoshuang Tang, Jin Zeng, Tie Chong, Xinyang Wang, Dalin He, Peng Guo

**Affiliations:** ^1^ Department of Urology The First Affiliated Hospital of Xi'an Jiaotong University Xi'an China; ^2^ Department of Urology The Second Affiliated Hospital of Xi'an Jiaotong University Xi'an China

**Keywords:** AMPK*α*, autophagy, bladder cancer, JQ1

## Abstract

**Aim:**

JQ1, a BET bromodomain inhibitor, is a promising therapeutic approach for bladder cancer (BC). Our study aimed to determine whether autophagy is induced by JQ1 and its potential role toward proliferation in BC.

**Methods:**

Cell proliferation was determined by methylthiazolyldiphenyl‐tetrazolium bromide (MTT) assay, cell counting assay, and colony formation assay. Autophagosomes and autolysosomes were observed by transmission electron microscopy and mRFP‐EGFP‐LC3 fluorescence assay. 3‐MA, BAFA1, NH_4_Cl, and siATG5 were used to inhibit autophagy. AMPK siRNA was used to knock down AMPK. T24 xenograft model in mice was chosen to perform in vivo studies. Autophagy markers LC‐3B and p62, p‐AMPK*α*, p‐ACC, p‐ULK1, p‐mTOR and p‐LKB1 were determined by western blot in vitro studies and by immunohistochemistry (IHC) in vivo specimens.

**Results:**

We found that BC cell proliferation was suppressed by JQ1; moreover, JQ1 induced the accumulation of autophagosomes and autolysosomes, and autophagy flux, and the growth suppression capacity of JQ1 was attenuated by autophagy inhibitors. Furthermore, we found that JQ1 induced the phosphorylation of AMPK*α*, and AMPK*α* knockdown attenuated autophagy induction and anti‐proliferation effect induced by JQ1 in BC cells, indicating that autophagy induced by JQ1 is dependent on AMPK*α*. Through endogenous immunoprecipitation analysis, we found that JQ1 dramatically increased the interaction between LKB1 and AMPK*α*, which may lead to more AMPK activation. Proliferation inhibition, autophagy induction, and LKB1/AMPK activation capacities of JQ1 were further confirmed in vivo.

**Conclusions:**

Taken together, our results demonstrate that autophagy is induced by JQ1 through activation of LKB1/AMPK pathway, and the autophagy induced by JQ1 positively contributes to the inhibition of BC cell proliferation. These findings provide a novel point of view to understand the mechanism of how targeting BET bromodomain suppress cancer cell growth and suggest that targeting BET bromodomain might be a potential approach to treat BC in the future.

## 1 INTRODUCTION

Bladder cancer (BC) is estimated to be the ninth most frequent cancer all over the world, and about 400 000 new cases are diagnosed each year.^1^, ^2^ Since the majority of BC diagnosed is as nonmuscle invasive bladder cancer (NMIBC), its mortality rates are generally lower compared to other cancers because of its favorable prognosis. However, NMIBC often recurs and progresses to muscle‐invasive bladder cancer (MIBC). With MIBC, the advanced‐stage of BC, the prognosis is quite poor mainly due to metastatic disease.^3^ Thus, there is an urgent need to identify potential therapeutic targets and develop their specific inhibitors or agonists to combat with BC as well as to prolong patients' survival.

Abundant evidence indicates that BET bromodomain, a family consists of BRD2, BRD3, BRD4, and BRDT, plays vital roles in the pathogenesis of specific leukemia as well as solid tumors, such as cervical cancer and bladder cancer.^4^, ^5^, ^6^ These proteins have been identified in several cellular programs contributing to neoplasia, including producing oncogenic fusion proteins and regulating the expression of tumor‐associated proteins, such as c‐Myc.^7^ Therefore, targeting BET proteins has emerged as a promising cancer therapeutic strategy. JQ1 is a novel small molecule that selectively targets and inhibits BET bromodomain, thereby suppressing the tumor through c‐Myc‐dependent and c‐Myc‐independent mechanisms.^8^, ^9^ Recent studies demonstrate that JQ1 inhibits BC cell proliferation via triggering apoptosis and inducing cell cycle arrest both in vitro and in vivo, which confirmed its anticancer potential toward BC.^10^ However, the underlying mechanism is not fully understood yet.

Autophagy plays a complicated role in cancers. On one hand, it prevents the accumulation of damaged proteins and organelles to suppress tumor activity; on the other hand, the process of autophagy provides tumor cells with materials like nutrition, which promotes tumor cell survival and enhances tumor growth.^11^ Recent research showed that ubenimex enhanced the anti‐glioma efficiency of JQ1 through suppressing autophagy.^12^ However, whether autophagy is induced by JQ1 in BC cells and whether autophagy modulates proliferation of BC cells is still unknown.

In the present study, we determined the autophagy induction capacity of JQ1 in BC and explored the effects of JQ1 on BC cell proliferation both in vitro and in vivo, moreover, we further identified whether autophagy induced by JQ1 modulates BC cell proliferation and the mechanism of how JQ1 induced autophagy, mainly focusing on LKB1/AMPK signal pathway.

## 2 MATERIALS AND METHODS

### 2.1 Reagents, antibodies and plasmids

DMEM culture medium, RMPI‐1640 culture medium, fetal bovine serum (FBS), and 100 × penicillin/streptomycin were purchased from HyClone Laboratories (Logan, UT). Detailed information of primary and secondary antibodies including names, manufacture, applications and dilutions are listed in Table 1. siAMPK*α*, siATG5, and control siRNA were purchased from Santa Cruz Biotechnology (Santa Cruz, CA). MTT, proteinase/phosphatase inhibitors cocktail, crystal violet, cyclohexane, and NH_4_Cl were purchased from Sigma‐Aldrich (St. Louis, MO). PVDF membrane and ECL reagents were purchased from Bio‐Rad Laboratories (Hercules, CA). Bafilomycin A1 (BAFA1) and 3‐Methyladenine (3‐MA) were purchased from EMD Millipore (Billerica, MA). DharmaFECT 1 transfection reagent was purchased from GE Healthcare Life Science (Marlborough, MA). The mRFP‐EGFP‐LC3 reporter plasmid (ptfLC3) was a gift from Dr Tamotsu Yoshimori (Addgene plasmid # 21074).^13^

**Table 1:** Antibodies used for western blotting analysis, IHC, and endogenous immunoprecipitation assays

Antibody	Source	Clone, Cat.#	Description	Application, dilution
p62	Cell Signaling Technology	88588	Mouse monoclonal	WB, 1:1000 IHC, 1:200
LC3‐B	Cell Signaling Technology	3868	Rabbit monoclonal	WB, 1:1000 IHC, 1:200
GAPDH	Santa Cruz	sc‐0411	Mouse monoclonal	WB, 1:10000
p‐LKB1 (Ser428)	Cell Signaling Technology	3482	Rabbit monoclonal	WB, 1:1000
p‐LKB1 (Ser428)	abcam	ab138386	Rabbit polyclonal	IHC, 1:300
LKB1	Cell Signaling Technology	3050	Mouse monoclonal	WB, 1:1000 IP, 1:100
p‐AMPK*α* (Thr172)	Cell Signaling Technology	50081	Rabbit monoclonal	WB, 1:1000 IHC, 1:200
AMPK*α*	Cell Signaling Technology	5832	Rabbit monoclonal	WB, 1:1000 IP, 1:100
p‐ACC (Ser79)	Cell Signaling Technology	11818	Rabbit monoclonal	WB, 1:1000
ACC	Cell Signaling Technology	3676	Rabbit monoclonal	WB, 1:1000
p‐mTOR (Ser2448)	Cell Signaling Technology	5536	Rabbit monoclonal	WB, 1:1000
p‐mTOR (Ser2448)	Cell Signaling Technology	2976	Rabbit monoclonal	IHC, 1:200
mTOR	Cell Signaling Technology	2983	rabbit monoclonal	WB, 1:1000
p‐ULK1 (Ser555)	Cell Signaling Technology	5869	Rabbit monoclonal	WB, 1:1000 IHC, 1:100
ULK1	Cell Signaling Technology	6439	Rabbit monoclonal	WB, 1:1000

### 2.2 Cell culture

T24, 5637, and UMUC‐3 cell lines were purchased from American Type Culture Collection (ATCC, Manassas, VA). T24 and UMUC‐3 were cultured in DMEM culture medium supplemented with 10% FBS, and 5637 was cultured in RMPI‐1640 culture medium supplemented with 10% FBS. Cells were incubated at 37˚C with the humidity set to 95%.

### 2.3 MTT assay

Cells (3000/well) were seeded into a 96‐well plate. After overnight incubation, culture medium was replaced with fresh medium supplemented with designated reagents (vehicle control, JQ1, 3‐MA and etc). After 48 hours, 200 μL 3‐(4, 5‐dimethylthiazol‐2‐yl)‐2, 5‐diphenyltetrazolium bromide (MTT) was added to each well to make a final concentration 0.5 mg/mL 2‐4 hours before harvesting. The medium aspirated carefully and then 150 μL DMSO was added to each well to dissolve the violet sediment. Afterward, the plate was gently vortexed for 5 minutes or until all the precipitation was dissolved. A Bio‐tek mirco‐plate reader (Winooski, VT) was utilized to read the optical density (OD) value with the wavelength set to 490 nm.

### 2.4 Cell counting assay

Cells (100 000/well) were seeded to the wells of a 6‐well plate. After overnight incubation, culture medium was replaced with fresh medium supplemented with designated reagents (vehicle control, JQ1, 3‐MA and etc). After 48 hours of treatment, cells were scraped and suspended, PBS was then added to make a final volume of 5 mL. The Beckman Coulter Z2 cell and particle counter (Indianapolis, IN, United States) was then used to determine the cell number.

### 2.5 Clone formation assay

Cells (100/well) were seeded to the wells of a 6‐well plate. After overnight incubation, culture medium was replaced with fresh medium supplemented with designated reagents (vehicle control, JQ1, 3‐MA and etc). After 2 weeks treatment, the medium was aspirated, the cells were rinsed with iced cold PBS for three times, and the cells were fixed within each well by adding 2 mL 4% paraformaldehyde. After 10 minutes fixation, cells were washed by ice‐cold PBS for three times. About 0.5% crystal violet staining (10 minutes) was then performed to make those cells visible. The wells were rinsed with PBS for three times, the solution was aspirated and then air‐dried in ambient temperature. The whole image of the plate was then captured using the Bio‐Rad ChemiDoc system (Hercules, CA).

### 2.6 GFP‐RFP‐LC3 fluorescence assay

Cells were seeded onto coverslip and incubated in the cell culture incubator overnight. Cells were then transient transfected with ptfLC3 expressing plasmid using Lipofectamine 2000 (Waltham, MA, USA) transfection system according to the manufacturer's instruction. After 24 hours, medium was aspirated and replaced with fresh medium supplemented with/without JQ1. After 24 hours of treatment, cells were fixed by 4% paraformaldehyde for 10 minutes and then rinsed three times with PBS. Mounting medium containing DAPI was dropped to the coverslip afterward followed by placing to a glass slide upside down. The localization of LC3 puncta was observed and captured by a fluorescence microscopy (×200, Olympus, Tokyo, Japan).

### 2.7 Transmission electron microscopy (TEM)

Cells (100 000/well) were seeded to the wells of a 6‐well plate. After overnight incubation, medium was aspirated and replaced with fresh medium supplemented with/without JQ1. After 24 hours treatment, cells were fixed in 4°C 2.5% glutaraldehyde in 0.01 mol/L PBS, pH 7.3 for 2 hours. Then cells were rinsed in PBS, post‐fixed in 1% osmium tetroxide with 1% potassium ferricyanide, dehydrated through a graded series of ethanol (30%‐90% and 100%‐Ethanol 200 Proof) and embedded in EPON. Semi‐thin (300 nm) sections were cut on Reichart Ultracut, stained with 0.5% toluidine blue and examined under a light microscope. Ultrathin sections (65 nm) were stained with 2% uranyl acetate and Reynold's lead citrate and examined on JEOL 1011 transmission electron microscope (JEOL, Peabody, MA).

### 2.8 Western blotting analysis

Cells were harvested using 1 × RIPA lysis buffer containing proteinase and phosphatase inhibitor cocktail and boiled with SDS loading buffer for 5 minutes. BCA quantification method was utilized to quantify protein concentration. Total protein from each sample (20 μg) was separated by 5%‐15% SDS‐PAGE. Proteins were then transferred to a PVDF membrane. After 1 hours blocking at room temperature or overnight blocking in 4°C in 5% skim milk, membranes were incubated with primary antibodies for 2 hours at room temperature or overnight at 4°C. Membranes were washed three times with TBST and then incubated with secondary antibodies for 1 hours at room temperature. After washing for three times in TBST, membranes were immersed into ECL mix for 5 minutes. Protein bands were detected using a Bio‐Rad ChemiDoc system (Hercules, CA) and quantified by Image J software (NIH, Bethesda, MD).

### 2.9 Reverse transcription‐quantitative polymerase chain reaction (RT‐qPCR)

Protocols used for mRNA isolation, cDNA reversing and RT‐qPCR were described previously.^14^ The following primers were used: p62: F 5′‐GCACCCCAAT‐GTGATCTGC‐3′; R 5′‐CGCTACACAAGTCGTAGTCTGG‐3′; GAPDH: F 5′‐CGACCACTTTGTCAAGCTCA‐3′; F 5′‐AGGGGAGATTCAGTGTGGTG‐3′.

### 2.10 siRNA transfection

siRNA was transfected into cells using DharmaFECT 1 transfection reagent (Lafayette, CO, USA) according to the manufacturer's protocol. Briefly, culture medium was replaced by serum‐free medium 1 hours before transfection. siRNA and transfection reagents were diluted in separately serum‐free medium and then mixed together and incubated at room temperature for 10 minutes. The transfection complex mixture was then added to the culture medium and mixed well by shaking back and forth several times. The medium was replaced by complete medium after 24 hours of incubation.

### 2.11 Endogenous immuno‐precipitation assay

Cells were harvested using lysis buffer (50 mm HEPES, pH 7.4, 100 mm NaCl, 1 mm EDTA, 1% Triton X‐100 and 1 mm PMSF) containing proteinase and phosphatase inhibitor cocktail. After 10 minutes centrifugation at 10 000 *g*, the supernatant was transferred to new EP tubes, incubated with antibodies overnight at 4°C with continuous low speed rotation, and incubated with Protein A conjugated agarose beads (Santa Cruz, Dallas, TX) for additional 1.5 hours with agitation. Then the beads were washed four times with lysis 1 × buffer, once with 20 mmol/L Tris‐HCl (pH 7.4), and finally boiled in 60 μL of 2 × SDS sample loading buffer for 5 minutes.

### 2.12 In vivo Xenografts

All animal studies were performed under the supervision and guidelines of the Institutional Animal Care and Use Committee of the Medical School, Xi'an Jiaotong University (Permission Number: SCXK2017‐0155, 5 March 2017). About 1 × 10^6^ T24 cells were injected subcutaneously into 20 4‐week‐old female nude mice. The animals were randomized into two groups (10 per group, vehicle control vs JQ1) 10 days after injection (tumors' volume was approximately 70 mm^3^), these animals were treated with JQ1 (50 mg/kg) or vehicle control once a day by intraperitoneal injection. A Vernier calipers was used to measure the two dimensions of the tumor every 3 days. The tumor volume was calculated as: volume = length × width^2^ × 0.5. After 2 weeks of treatment, animals were sacrificed. The tumors were weighted, fixed in 4% paraformaldehyde, and embedded in paraffin. Proteins expression of interest was determined by Immunohistochemistry (IHC).

### 2.13 Immunohistochemistry (IHC) assay

IHC was performed using rabbit ABC staining system according to the manufacturer's manual (Santa Cruz, Dallas, TX). Formalin‐fixed paraffin‐embedded (PFPE) sample slides were deparaffined and dewatered first. Tris‐based antigen unmasking solution (Vector laboratories, Burlingame, CA) was utilized to perform antigen retrieval. Endogenous peroxidase activity was quenched by 3% hydrogen peroxide diluted in PBS for 30 minutes at room temperature. Sections were incubated with 1.5% blocking serum diluted in PBS for 1 hours at room temperature, incubated with primary antibody overnight at 4°C and secondary antibody at room temperature for 30 minutes. Primary antibodies are listed in Table 1. Subsequently, sections were incubated with AB enzyme mix for 30 minutes and then DAB peroxidase substrate for 3 minutes at room temperature (Vector laboratories, Burlingame, CA). Counterstained sections in Gill's formulation hematoxylin for 15 seconds and dehydrated sections afterward. One to two drops of permanent mounting medium were added to sections and were covered with glass coverslips.

### 2.14 Statistical analysis

All statistical graphs were generated by GraphPad Prism 7 software (GraphPad Software, Inc La Jolla, CA). All experiments were performed at least three times. Student's *t* test or post hoc test used with ANOVA was utilized to make statistical analysis between or among groups. A *P* < 0.05 was considered to be statistically significant.

## 3 RESULTS

### 3.1 JQ1 inhibits proliferation of BC cells

**Figure 1:**
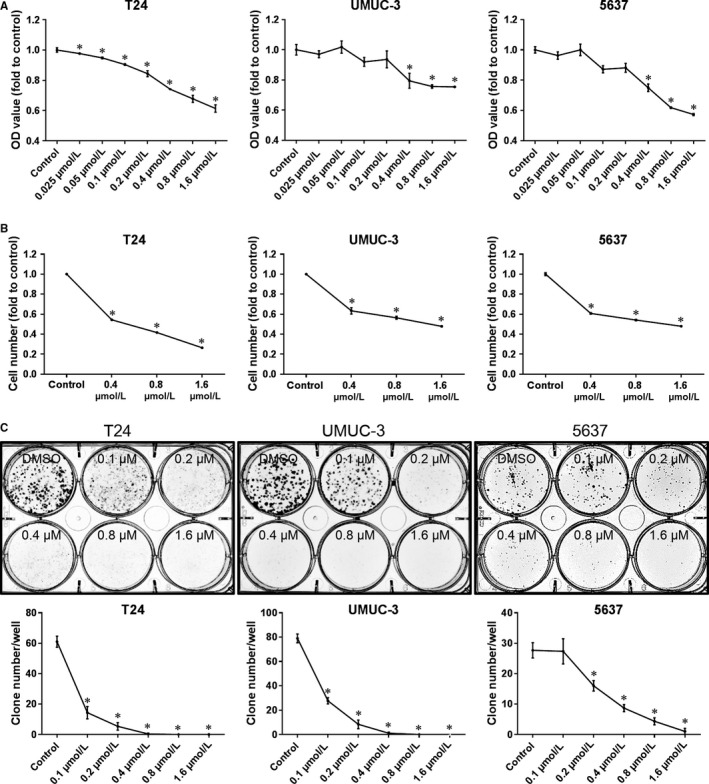
JQ1 inhibits cell proliferation in BC cells. After 48 h of treatment, cell proliferation was determined by MTT assay (A) and cell counting assay (B). The long‐term influence of JQ1 treatment on BC proliferation was studied by clone formation assay (C). *: *P* < 0.05 vs control. Data represent the results of three independent experiments

JQ1, a BET bromodomain inhibitor, has been reported to inhibit the proliferation of BC cells.^10^ To further confirm these results, we determined the effect of JQ1 on T24, UMUC‐3 and 5637 BC cell lines. As shown in Figure 1, after 48 hours of treatment, proliferations of these cells were suppressed by JQ1 in a dose‐dependent manner in MTT assay (Figure 1A) and cell counting assay (Figure 1B). To determine the long‐term influence of JQ1 on BC cell proliferation, clone formation assay was performed. As shown in Figure 1C, JQ1 treatment significantly decreased the numbers of clones formed in a dose‐dependent manner. These results demonstrate the anti‐proliferation ability of JQ1 toward BC cells.

### 3.2 JQ1 induces autophagy phenotypes in BC cells

**Figure 2:**
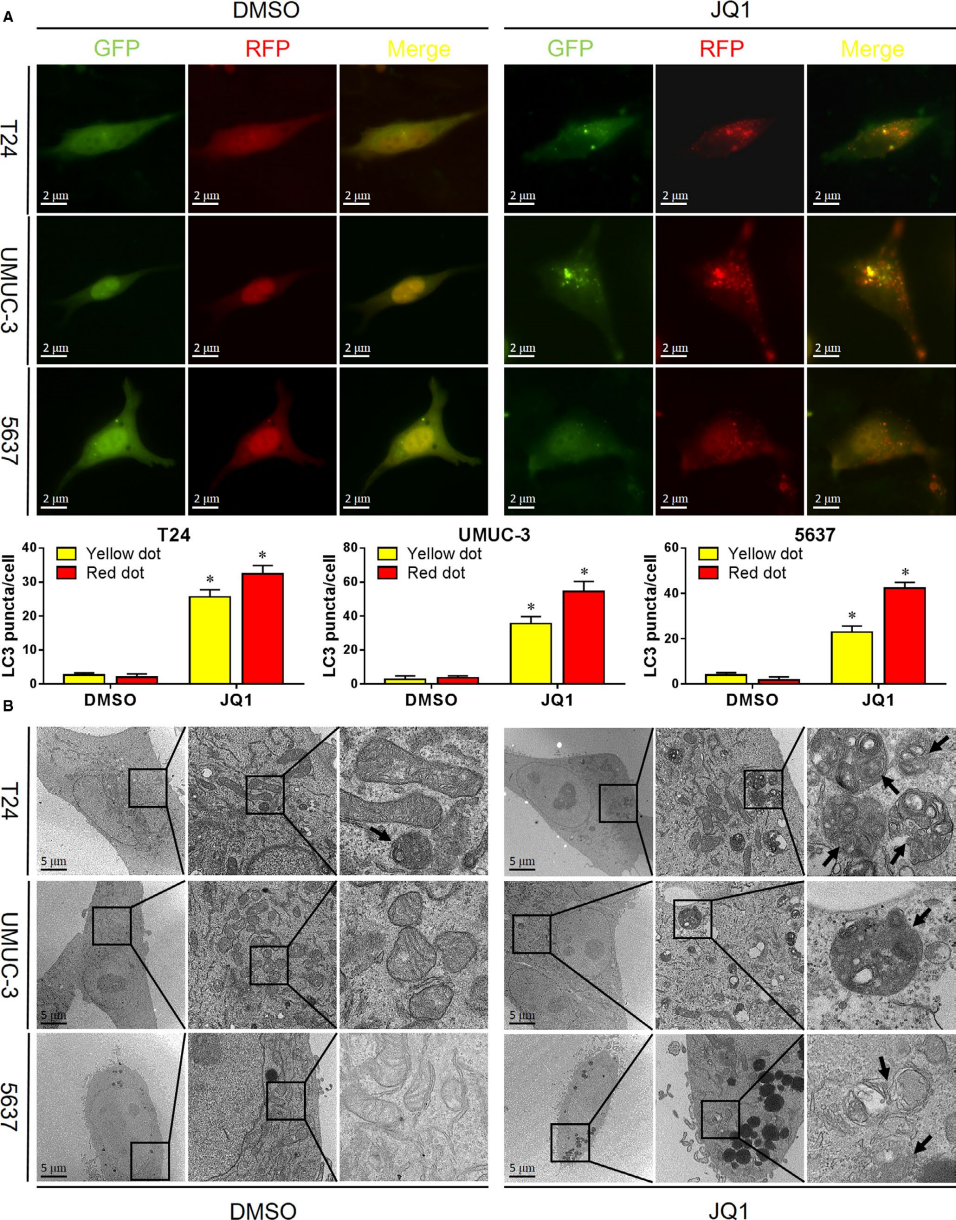
JQ1 induces autophagy in BC cells. A. Cells were transiently transfected with GFP‐RFP‐LC3 plasmid. After 24 h, cells were treated with JQ1 (0.8 μmol/L) for additional 24 h. Autophagy‐like red dots and yellow dots were observed by a fluorescence microscopy and were statistically analyzed. B. Autophagy‐like ultrastructures were observed under the TEM after 24 h treatment of JQ1 (0.8 μmol/L). Rectangle indicates the 4‐fold magnified image. Black arrows point to autophagy‐like ultrastructures

In BC, cell proliferation was inhibited by JQ1.^10^ However, whether autophagy, which also plays a pivotal role in proliferation modulation, is induced by JQ1 is unknown. As shown in Figure 2A, JQ1 treatment significant increased the red dots and yellow dots in GFP‐RFP‐LC3 fluorescence assay compared to vehicle control, indicating the induction of cell autophagy. To further confirm this finding, transmission electron microscopy (TEM), which is a gold standard of autophagy evaluation, was utilized to observe the ultrastructure of cells treated with/without JQ1. As presented in Figure 2B, JQ1 treatment induced significant more autophagic double‐membrane compartments containing lamellar structures compared to vehicle control. These results indicate the induction of autophagy by JQ1 in BC cells.

### 3.3 JQ1 induces autophagic flux in BC cells

**Figure 3:**
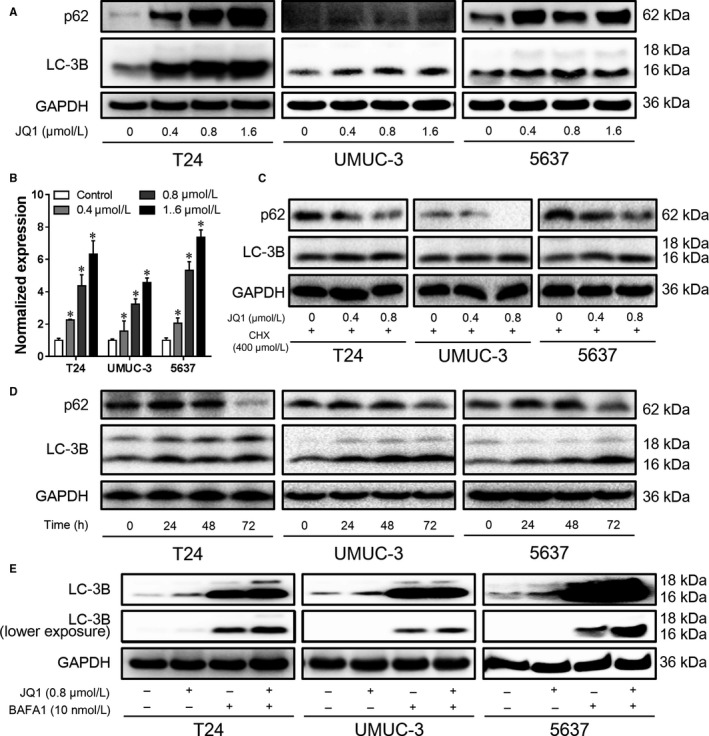
JQ1 induces autophagy flux in BC cells. After 24 h treatment, the expression of p62 and LC‐3B was detected by western blotting analysis (A), the mRNA level of p62 was also determined by RT‐qPCR assay (B). (C) Cells were pretreated with 400 nmol/L cyclohexane (CHX) for 4 h, and then treated with JQ1 in the presence of 400 nmol/L CHX. The expression of p62 and LC‐3B was detected by western blotting analysis. (D) Cells were treated with 0.4 μmol/L JQ1 for different time points, the expression of p62 and LC‐3B was detected by western blotting analysis. (E) Cells were treated with 0.8 μmol/L JQ1 for 12 h, then the culture medium was replaced with fresh medium containing 0.8 μmol/L JQ1 and 10 nmol/L BAFA1. After 12 h, cells were harvested and the expression of LC‐3B was detected by western blotting analysis. *P < 0.05 vs control. Data represent the results of three independent experiments

To further confirm the autophagy induced by JQ1, autophagy marker LC‐3B and autophagy substrate p62 were determined by western blotting analysis after 24 hours treatment with JQ1. We found that JQ1 treatment significantly increased the expression of LC‐3B, suggesting the induction of autophagy. To our surprise, p62, which was expected to be downregulated, was increased in JQ1 treatment (Figure 3A). Since p62 could be induced in the process of autophagy,^15^ to test this possibility, p62 mRNA expression was determined by RT‐qPCR assay. Just as expected, JQ1 treatment significantly upregulated the expression of p62 mRNA (Figure 3B). To exclude the confounding factor of p62 upregulation, we first inhibited the expression of p62 by cyclohexane and then checked the impacts of JQ1 on p62 expression. As shown in Figure 3C, with the treatment of cyclohexane, the expression of p62 was dramatically downregulated. To determine the long‐term effects of JQ1 on these two autophagy markers, we treated cells with JQ1 in a time‐course manner. As shown in Figure 3D, LC‐3B accumulated over time as expected. The expression of p62 was upregulated up to 48 hours, but in 72 hours, it was downregulated to a level lower than the control. Moreover, to exclude the contribution of autophagy blockage to LC‐3B elevation, we performed LC‐3 turnover assay. As presented in Figure 3E, the expression of LC‐3B was upregulated by JQ1 or BAFA1 alone and was enhanced when JQ1 and BAFA1 were combinedly used. These findings demonstrate that autophagy flux is induced by JQ1 in BC.

### 3.4 Autophagy induced by JQ1 contributes to proliferation inhibition

**Figure 4:**
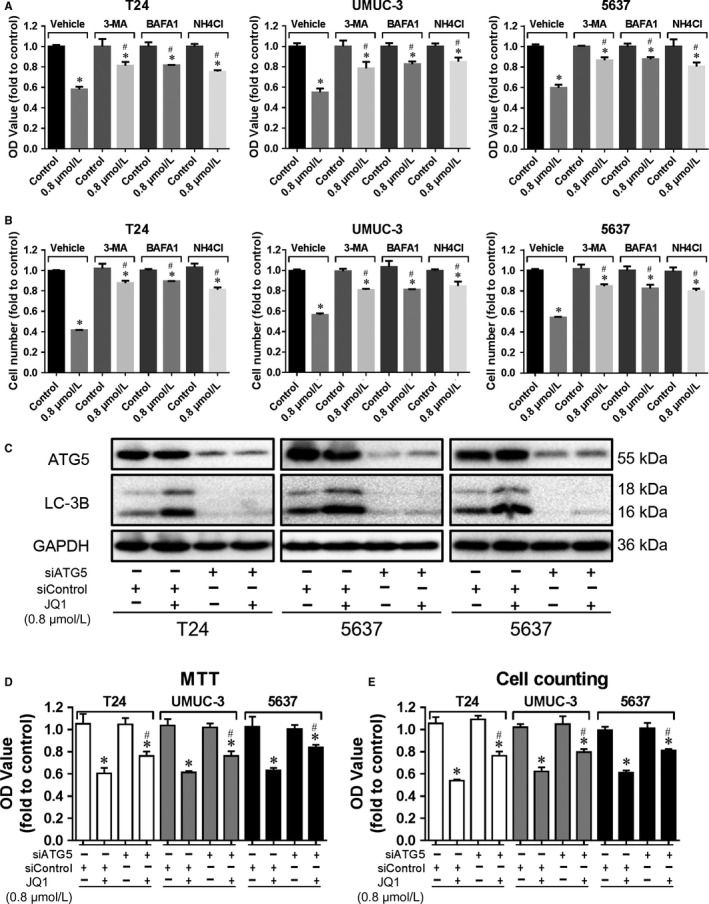
Autophagy induced by JQ1 contributes to proliferation inhibition. Cells were treated with JQ1 (0.8 μmol/L) with/without the presence of vehicle, 3‐MA (5 mmol/L) or BAFA1 (10 nmol/L) or NH4Cl (10 mmol/L) for 48 h, then cell proliferation was determined by MTT assay (A) and cell counting assay (B). C, D, E. Cells were transiently transfected with siATG5 or siControl. After 24 h, cells were treated with JQ1 (0.8 μmol/L) for additional 24 h. The expression of p‐ATG5 and LC‐3B was checked by western blotting analysis (C), cell proliferation was evaluated by MTT assay (D) and cell counting assay (E). *P < 0.05 vs control, ^#^ *P* < 0.05 vs JQ1 in the vehicle group. Data represent the results of three independent experiments

**Figure 5:**
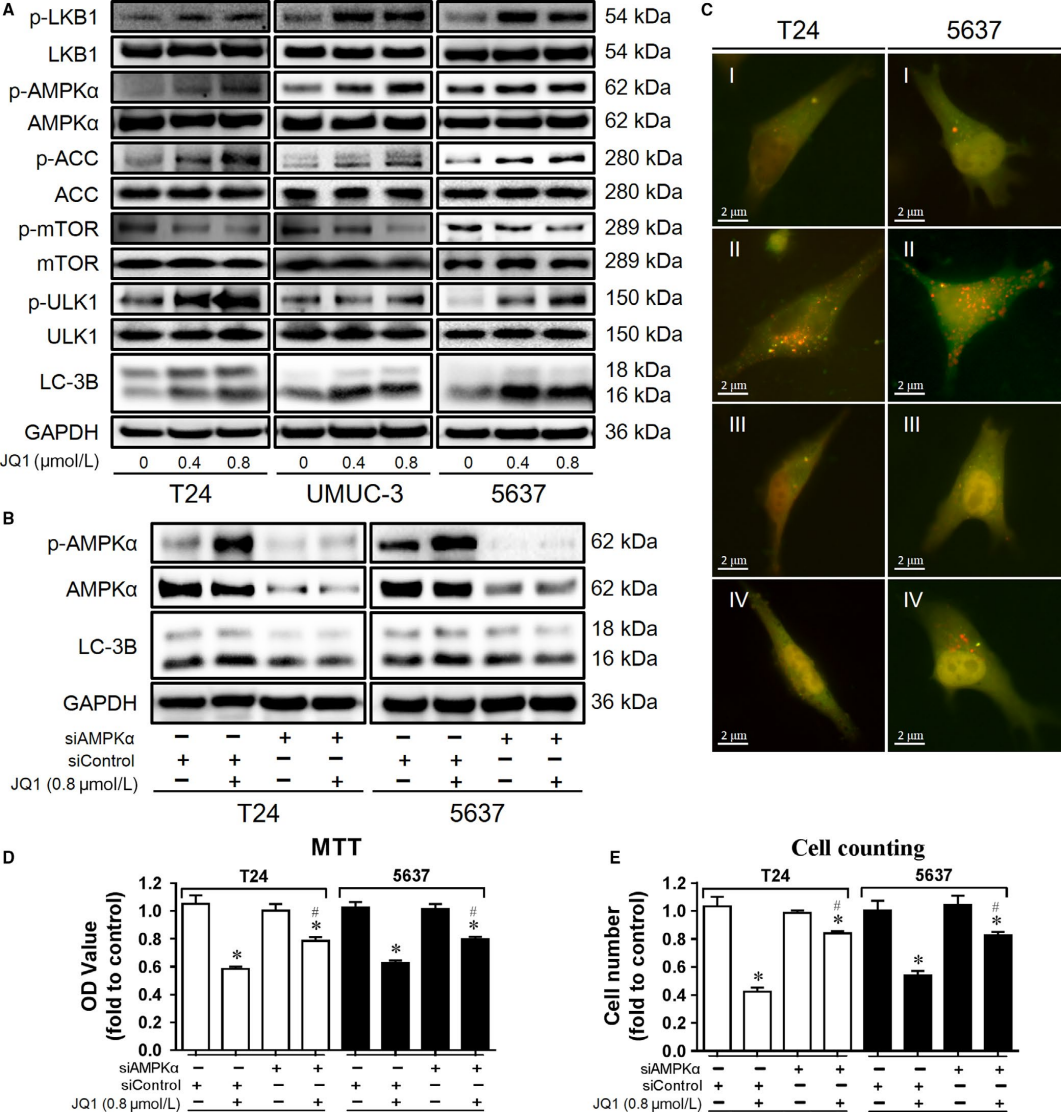
JQ1‐inducing autophagy is dependent on LKB1/AMPK/mTOR signal pathway. (A) After 24 h treatment with JQ1 (0.8 μmol/L), the expression of autophagy‐related proteins was checked by western blotting analysis. B, C, D, E. Cells were transiently transfected with siAMPK*α* or siControl. After 24 h, cells were treated with JQ1 (0.8 μmol/L) for addition 24 h. The expression of p‐AMPK*α*, AMPK*α* and LC‐3B was checked by western blotting analysis (B), autophagy‐like red dots and yellow dots were observed by a fluorescence microscopy (I, siControl + control; II, siControl + JQ1; III, siAMPK*α* + control; IV, siAMPK*α* + JQ1) (C), cells proliferation was evaluated by MTT assay (D) and cell counting assay (E). *P < 0.05 vs control, ^#^ *P* < 0.05 vs JQ1 in the vehicle group. Data represent the results of three independent experiments

Autophagy is confirmed to be induced by JQ1 in BC, however, whether the induced autophagy has impact on cell proliferation remains to be elucidated. As determined by MTT assay (Figure 4A) and cell counting assay (Figure 4B), cell proliferation was inhibited by JQ1 treatment, however, the proliferation inhibition capacity of JQ1 was attenuated by autophagy inhibitors 3‐MA, BAFA1 or NH_4_Cl. Similar results were observed when autophagy was suppressed by interfering critical autophagy regulator ATG5 using its specific siRNA (Figures 4C & 5D). These results indicate that autophagy induced by JQ1 positively contributes to the inhibition of cell proliferation in BC.

### 3.5 JQ1‐inducing autophagy is dependent on LKB1/AMPK/mTOR signaling pathway

To further understand the underlying mechanisms of JQ1‐inducing autophagy, the expression of key autophagy‐related proteins was determined by western blotting analysis. As shown in Figure 5A, JQ1 treatment increased the expression of LC‐3 B and p‐ULK1, which further confirmed the induction of autophagy. Moreover, p‐mTOR was downregulated while p‐LKB1, p‐AMPK*α* and its substrate p‐ACC were upregulated by JQ1 treatment, indicating that LKB1/AMPK/mTOR signaling was involved in JQ1 induced autophagy. To detect the function of AMPK*α* in JQ1 induced autophagy, AMPK*α* was knocked down by specific AMPK*α* siRNA. We found that the expression of LC‐3 B as well as the number of red and yellow dots were increased by JQ1, while that increases were attenuated by AMPK*α* knockdown, as detected by western blotting analysis and GFP‐RFP‐LC3 fluorescence assay (Figure 5B & 5C). In addition, the inhibition capacity of JQ1 on cell proliferation was also attenuated by AMPK*α* knockdown (Figure 5D & 5E). Taken together, these results indicate that autophagy induced by JQ1 is dependent on LKB1/AMPK/mTOR signaling pathway.

### 3.6 JQ1 treatment increases the interaction between LKB1 and AMPKα

**Figure 6:**
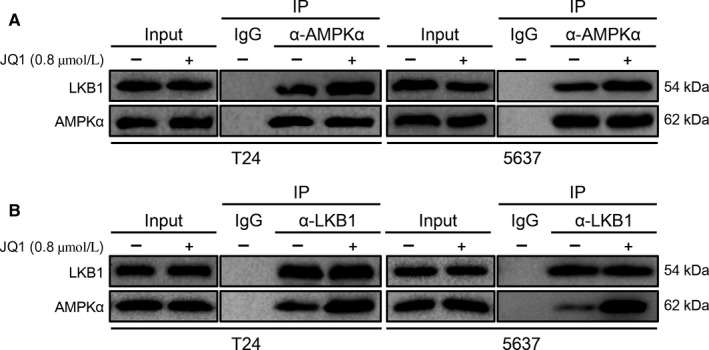
JQ1 treatment increases the interaction between LKB1 and AMPK*α*. After 24 h of treatment with JQ1 (0.8 μmol/L), AMPK/LKB1 complex was pulled down by anti‐AMPK*α* (A) or anti‐LKB1 (B), the expression of AMPK*α* and LKB1 was checked by western blotting analysis

Since JQ1 treatment did not affect the expression of total AMPK*α* and LKB1 but significantly increased p‐AMPK*α* and p‐LKB1 level, and LKB1 is one of important upstream activators of AMPK*α*, we deduce that JQ1 treatment may increase the recruitment of LKB1 to bind AMPK*α* and then activate it. To test this hypothesis, we performed endogenous immunoprecipitation in T24 and 5637 BC cells, and found that JQ1 treatment obviously increases the interaction between LKB1 and AMPK*α* (Figure 6A), and vice versa (Figure 6B). These findings suggest that JQ1 may induce AMPK activation by increasing the interaction between LKB1 and AMPK*α*.

### 3.7 JQ1 inhibits BC growth and increases cell autophagy in vivo

**Figure 7:**
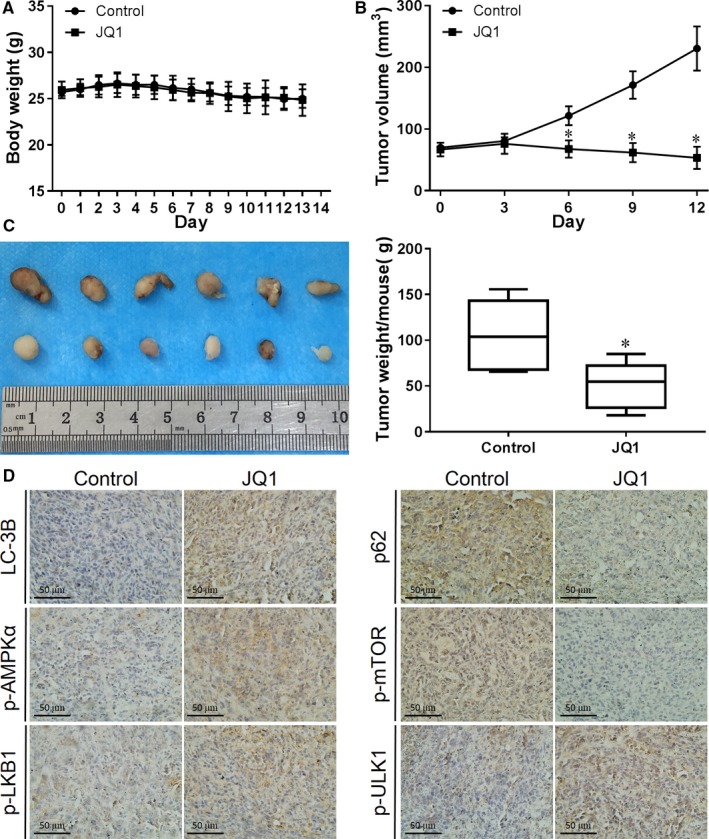
JQ1 inhibits BC growth and increases cell autophagy in vivo. Tumor bearing mice were treated with JQ1 (50 mg/kg) or with vehicle control once a day by intraperitoneal injection. Mice' body weight was checked every day (A), tumor size was measured every 3 days (B). Tumors were harvested after 2 weeks of treatment, then images were taken (C) and tumors were weighted (D). The expression of LC‐3B, p62, p‐AMPK*α*, p‐mTOR, p‐LKB1 and p‐ULK1 was determined by IHC. *P < 0.05 vs control

To determine the antitumor and autophagy induction capacities of JQ1 in vivo*,* we injected T24 BC cells subcutaneously into nude mice and made xenograft tumor model, and then treated mice with JQ1 for 2 weeks. We found that JQ1 had no effect on mice body weight comparing to vehicle control (Figure 7A), however, both tumor volume (Figure 7B) and weight (Figure 7C) were significantly inhibited by JQ1. LC3‐B and p‐ULK1 were upregulated while p62 was downregulated in JQ1‐treated mice comparing to the vehicle control (Figure 7D), indicating the induction of autophagy. Consistent with the in vitro study, JQ1 treatment increased the expression of p‐AMPK*α* and p‐LKB1 but downregulated p‐mTOR in vivo, further confirming the regulation of LKB1/AMPK/mTOR signal pathway by JQ1 (Figure 7D). Taken together, these results indicate that JQ1 treatment inhibited BC growth, increased cell autophagy, and activated LKB1/AMPK signaling in vivo.

## 4 DISCUSSION

JQ1 was shown to inhibit proliferation of bladder cancer cells^10^; however, the underlying mechanisms, such as whether autophagy is induced by JQ1 in BC cells and whether the autophagy modulates the proliferation of BC cells are still unknown. Herein, in BC cells, we show for the first time that autophagy was induced by JQ1 and autophagy positively contributed to the inhibition of cell proliferation both in vitro and in vivo; moreover, JQ1‐inducing autophagy is dependent on the activation of LKB1/AMPK signaling. These results indicate that autophagy induction plays an important role for the cell proliferation inhibitory effect of JQ1 on BC cells, which might provide a novel point of view to understand the mechanism of how JQ1 suppresses cancer cell growth.

Autophagy is a double‐edged sword, with one edge of tumor promoter and the other edge of tumor suppressor.^11^, ^16^ Autophagy induction may contribute to inhibit cell growth, however, autophagy might also protect some cancer cells against chemotherapy or radiotherapy.^11^, ^17^, ^18^ Thus, the exact effect of autophagy on cancer proliferation may be context‐dependent. Firstly, we detected whether JQ1 induced autophagy in BC cells. In GFP‐RFP‐LC3 fluorescent assay, JQ1 treatment increased yellow dots and red dots, which represented autophagosomes and autophagolysosomes respectively. Moreover, these autophagic vacuoles were confirmed to be autophagic double‐membrane compartments containing lamellar structures under the TEM. Next, these phenotypes were further confirmed in LC‐3 turnover assay and p62 turnover assay. These results demonstrate that JQ1 induced autophagy flux in BC cells. On the other hand, proliferation of BC cells was significantly inhibited by JQ1 treatment. However, when autophagy was suppressed pharmaceutically by 3‐MA, BAFA1 and NH_4_Cl, which are autophagy inhibitors, as well as genetically by siATG5, the inhibitory ability of JQ1 on cell proliferation was attenuated. Taken together, these results indicate that autophagy induction positively contributes to cell growth suppression by JQ1. It was noted that, with autophagy inhibition, JQ1 could still partly inhibit the proliferation of BC, suggesting that other cell death programs like cell apoptosis and/or cell cycle arrest might be involved in the cell proliferation inhibition.

To further investigate the underlying mechanism of how JQ1 induced autophagy in BC cells, we detected the status of autophagy‐related upstream signaling, focusing on AMPK/mTOR signal pathway. With JQ1 treatment, p‐AMPK*α* and its substrate p‐ACC were upregulated while p‐mTOR was downregulated, suggesting that AMPK/mTOR signaling was regulated by JQ1. Total AMPK*α* stayed unchanged while p‐AMPK*α* was significantly upregulated, which indicate that JQ1 regulates AMPK*α* through its phosphorylation rather than its protein expression. We found that AMPK activation was essential for JQ1‐induced autophagy and proliferation suppression, because both of them were attenuated when AMPK*α* was knocked down by its specific siRNA. Moreover, it is notable that expression of p‐LKB1, a direct upstream activator of AMPK*α*, was upregulated by JQ1, indicating the involvement of LKB1/AMPK signaling. This hypothesis was further confirmed by endogenous immunoprecipitation assay. We found that, with JQ1 treatment, more LKB1 was recruited to bind AMPK*α* and thus lead to its activation. Nevertheless, AMPK*α* is regulated by a complicated network, thus whether other factors rather than LKB1 are also involved is unknown. Recent studies show that JQ1 synergizes with PARP inhibitor to increase DNA damage in epithelial ovarian cancer.^19^ DNA damage and metabolism are connect by the crosstalk between PARP1 and SIRT1, a potent activator of AMPK*α*.^20^ Therefore, it will be intriguing to explore the participation of PARP/SIRT1/AMPK signaling in JQ1 induced autophagy in the future.

JQ1 selectively targets and inhibits BET bromodomain, and numerous studies have reported that it suppresses tumor growth through c‐Myc‐dependent and c‐Myc‐independent mechanisms.^8^, ^9^ In the present study, we found that JQ1 induces the activity of LKB1/AMPK pathway and autophagy in BC cells, which contributes to the cell proliferation inhibition. This may be happen with downregulation of the c‐Myc and its target genes, such as EZH2, and changes of c‐Myc‐independent pathways simultaneously, which indicating the complexity of how JQ1 inhibits cell proliferation. Considering this autophagy induction function of JQ1, the application of combination JQ1 with chemotherapy and radiotherapy may be limited because autophagy might result in cell resistance to other stress.^21^, ^22^

The proliferation inhibition and autophagy induction abilities of JQ1 in BC as well as the involvement of LKB1/AMPK/mTOR signaling were further confirmed by utilizing T24 cell xenografts in nude mice. However, considering the long‐term inhibition/activation efficiency and the cytotoxicity of autophagy inhibitors and AMPK inhibitors on tumor cells and their side effects on tumor‐bearing animals, our in vivo studies did not apply these chemicals on animals.^23^, ^24^ In the future, LKB1 or AMPK genetically knockdown or knockout animals may be used to detect whether LKB1/AMPK is essential for JQ1 to induce autophagy and inhibitory BC cell proliferation in vivo, though whether the knockdown/knockout may directly change cell proliferation is still a concern. It is noted that the protein level of p62 was decreased in vivo, which seems to be conflict to what was observed in vitro. As JQ1 both activates p62 transcription and increases its posttranslational autophagic degradation, the balance between them determines the intracellular level of p62.^25^ One explanation is that, in short‐term treatment in vitro, more p62 is induced because of stress; however, in long‐term exposure in vivo, autophagic degradation takes the dominate role as cells are adapted to this kind of stress. In vitro studies, we do see a trend of p62 downregulation after 72 hours of treatment with JQ1, however, more efforts are needed to fully get it answered in the future.

Taken together, our present results demonstrate that autophagy is induced by JQ1 in BC cells through the activation of LKB1/AMPK pathway, and the autophagy induced by JQ1 positively contributes to the inhibition of BC cell proliferation. These findings provide a novel point of view to understand the mechanism of how JQ1 suppress cancer cell growth and suggests that JQ1 might be a potential chemotherapeutic for BC in the future.

## CONFLICTS OF INTEREST

The authors declare no conflict of interest.

## AUTHOR CONTRIBUTIONS

DH and PG designed and supervised the study. FL, CY, JM and JZ performed the in vitro assays. HZ and JJ managed the animal experiment. XT contributed to IHC staining and manuscript improvement. TC and XW analyzed the data. FL and PG wrote the manuscript. All authors read and approved the final manuscript.
